# Long-Term Weight Change after Initiating Second-Generation Antidepressants

**DOI:** 10.3390/jcm5040048

**Published:** 2016-04-13

**Authors:** David Arterburn, Tamar Sofer, Denise M. Boudreau, Andy Bogart, Emily O. Westbrook, Mary Kay Theis, Greg Simon, Sebastien Haneuse

**Affiliations:** 1 Group Health Research Institute, 1730 Minor Ave, Suite 1600, Seattle, WA 98101, USA; boudreau.d@ghc.org (D.M.B.); abogart@rand.org (A.B.); westbrook.e@ghc.org (E.O.W.); theis.m@ghc.org (M.K.T.); simon.g@ghc.org (G.S.); 2 University of Washington, UW Tower, 15th Floor, 4333 Brooklyn Ave NE, Seattle, WA 98105, USA; tsofer@uw.edu; 3 Harvard T.H. Chan School of Public Health, 677 Huntington Ave, Boston, MA 02115, USA; shaneuse@hsph.harvard.edu

**Keywords:** obesity, antidepressant, depression, adverse effects, weight gain

## Abstract

(1) Objective: To examine the relationship between the choice of second-generation antidepressant drug treatment and long-term weight change; (2) Methods: We conducted a retrospective cohort study to investigate the relationship between choice of antidepressant medication and weight change at two years among adult patients with a new antidepressant treatment episode between January, 2006 and October, 2009 in a large health system in Washington State. Medication use, encounters, diagnoses, height, and weight were collected from electronic databases. We modeled change in weight and BMI at two years after initiation of treatment using inverse probability weighted linear regression models that adjusted for potential confounders. Fluoxetine was the reference treatment; (3) Results: In intent-to-treat analyses, non-smokers who initiated bupropion treatment on average lost 7.1 lbs compared to fluoxetine users who were non-smokers (95% CI: −11.3, −2.8; *p*-value < 0.01); smokers who initiated bupropion treatment gained on average 2.2 lbs compared to fluoxetine users who were smokers (95% CI: −2.3, 6.8; *p*-value = 0.33). Changes in weight associated with all other antidepressant medications were not significantly different than fluoxetine, except for sertraline users, who gained an average of 5.9 lbs compared to fluoxetine users (95% CI: 0.8, 10.9; *p*-value = 0.02); (4) Conclusion: Antidepressant drug therapy is significantly associated with long-term weight change at two years. Bupropion may be considered as the first-line drug of choice for overweight and obese patients unless there are other existing contraindications.

## 1. Introduction

Obesity and depression are major public health concerns with substantial impacts on medical morbidity, health care spending, and quality of life [1,2,3,4,5,6,7]. In 2011–2012, the prevalence of obesity (defined as a body mass index [BMI] of ≥30 kg/m^2^) among US adults was 34.9% [8], and, in 2008, 10.4% of US adults were taking antidepressant medications [9]. Obesity and depression also commonly occur together, and adults with both conditions may have even greater health risks [10,11,12]. The causal pathway is probably bidirectional—obese adults are at greater risk of depression and *vice versa*.

With climbing rates of obesity [13,14] and antidepressant agents now the most commonly prescribed drugs in the US [15], the potential impact of antidepressant use on obesity risk is receiving renewed attention [16]. There is now a growing body of evidence that the choice of antidepressant drug therapy may influence changes in weight [17,18]. Previous studies indicate that certain antidepressants (e.g., fluoxetine and bupropion) may cause weight loss, while others (e.g., paroxetine and mirtazapine) may cause weight gain, although follow-up data is generally limited to the first 12 months after treatment [18,19,20,21,22,23,24]. Less evidence is available regarding the long-term impact of antidepressants on weight, and some associations appear to be transient [18,19,20,21,22,23]. Still, many patients are prescribed antidepressants for long time periods, and it is important to know whether this longer-term exposure is related to weight gain.

Second-generation antidepressants (*i.e.*, selective serotonin reuptake inhibitors (SSRIs), serotonin norepinephrine reuptake inhibitors (SNRIs), and selective serotonin norepinephrine reuptake inhibitors (SSNRIs)) are the most commonly prescribed medications for primary treatment of depression, and they have similar efficacy and lower toxicity in overdose than first-generation antidepressants [25]. Current evidence indicates that second-generation antidepressants do not differ from each other on the basis of efficacy [26,27]. Therefore, treatment guidelines suggest that clinicians should select antidepressants on the basis of adverse effect profiles, costs, and patient preferences [25]. If antidepressants differ significantly in their long-term impact on weight, then patients and clinicians could preferentially choose those medications that are associated with the least amount of weight gain when prescribing them for patients who are normal weight, overweight, or obese. Thus, having more information on the long-term impact of antidepressants on weight could reduce the downstream risk of weight-related morbidity for a large population of adults with depression.

The goal of this study was to examine the relationship between the choice of antidepressant drug treatment and long-term weight gain at the population level. Because one of the most commonly prescribed antidepressants, bupropion, is also used as an adjunct to smoking cessation, and smoking cessation is strongly associated with weight gain [28], we sought to examine the effects of this antidepressant on weight gain among smokers and non-smokers separately. To accomplish these goals, we conducted a retrospective electronic health record (EHR)-based cohort study of patients initiating a monotherapy second-generation antidepressant (hereafter referred to as “antidepressant”) drug treatment between 2005 and 2009.

## 2. Experimental Section

### 2.1. Setting

The study was conducted at Group Health (GH), an integrated health plan and care delivery system that provides comprehensive health care to approximately 600,000 individuals in Washington State and Idaho. Among these enrollees, 65% receive care at GH Integrated Group Practice (IGP) clinics while the remaining enrollees are allied with non-GH provider networks where the primary source of data is the claims system. Information on health plan enrollment and health care use including diagnoses, procedures, pharmacy dispensings, and laboratory values are recorded and maintained in GH’s automated electronic databases. Further, since 2005, a fully-integrated EHR system documents all patient care at GH IGP clinics.

GH provides specialty mental health care to its enrollees using a combined IGP/network model. GH guidelines emphasize cognitive-behavioral therapy as part of first-line therapy for depressive disorders; however, antidepressant drug treatment surpasses psychotherapy as the initial treatment of choice for depression at GH, and 75% of antidepressant therapy is prescribed by Primary Care Physicians (PCPs).

The GH population closely resembles the underlying Washington state community with respect to age, race, and gender [29]. GH insurance plans vary considerably in the level of cost-sharing for outpatient primary care and mental health care, but copayments for routine outpatient care and psychotherapy are similar to national averages. Prior studies suggest that GH enrollees obtain approximately 97% of their prescription medications at GH owned and contracted pharmacies [29,30,31].

### 2.2. Study Population

The study population consists of adults aged 18–65 years with a diagnosis of depressive disorder (International Classification of Diseases, 9th Revision, ICD-9: 296.2×, 296.3×, 311, or 300.4) and who initiated a new monotherapy episode of antidepressant drug treatment. The latter was defined as a dispensing episode for a single antidepressant medication, without any other antidepressant medication dispensing in the prior nine months. The date of initiation/prescription is referred to as the “baseline date”, Our study sample was restricted to subjects who initiated the new treatment episode between 1 January, 2006 and 31 October, 2009, and had at least nine months of continuous enrollment in the health system prior to their baseline date.

Patients were excluded if they were taking medications or underwent procedures that have a strong effect on weight change. These included second/third-generation antipsychotic medications, lithium, valproate, weight loss medications, oral steroids, bariatric surgery, and dialysis. A full list of all excluded medications is provided in Table S1. Similarly, patients with histories of cirrhosis, eating disorder, pregnancy, or dementia at baseline were excluded from our analyses based on the presence of diagnosis codes.

### 2.3. Data Collection

Demographic and enrollment information, weight and height, prescription medication use, health care encounters, and medical conditions were extracted from GH electronic health care databases for all years of our study.

### 2.4. Antidepressant Drug Treatment

We extracted all fills for second-generation antidepressant medications, the primary exposure of interest, from 04/2005–09/2010. These included fluoxetine, citalopram, bupropion, paroxetine, sertraline, trazodone, mirtazapine, venlafaxine, and duloxetine. During the years of our study the second-generation antidepressant medications ecitalopram, fluvoxamine, and nefazodone were not on the Group Health formulary. As a result these three medications were rarely prescribed and were excluded from our analyses due to low sample sizes. A full list of all included and excluded medications is provided in Table S1.

### 2.5. Weight Data

The primary outcome of interest was the change in weight between the start of the new treatment episode (baseline) and two years post-baseline. Weight data was extracted from all encounters from 04/2005–09/2010. Encounters were classified according to several criteria: (i) occurring within the IGP *vs.* with a network provider; (ii) ambulatory (*i.e.*, in-person, outpatient) encounter *vs.* other encounter type (emergency room and inpatient); and (iii) primary care *vs.* specialty visit. In the GH IGP, care standards indicate that weights should be obtained at each outpatient visit without extra clothing and shoes. Prior research at GH indicates that weight measures routinely obtained in clinical care are highly correlated with those obtained by trained research staff and may be used in research studies without statistical correction [32].

To account for missingness in the weight data, we adopted a strategy that combined imputation prior to the main statistical analyses with inverse probability weighting in the main statistical analyses (see below). Prior to imputation we applied a novel cleaning algorithm that removed implausible measurements (*i.e.*, those outside 50–500 lbs.) as well as measurements that were inconsistent with an individual’s weight trajectory. Briefly, this algorithm consisted of fitting an unadjusted linear model of weight as a function of time to each subject’s weight data separately, and excluding those measurements for which the standardized residual was greater than 3, provided the subject had enough weight measurements to estimate such a model. For subjects with too few weight measurements to fit the linear model, no measurements were excluded. 

At baseline, we adopted the following sequential imputation strategy for weight: (i) if weight information was available on the baseline date, we took that value; (ii) otherwise, if a weight measurement was observed in the 30 days prior to the baseline date, we carried that value forward; (iii) otherwise, weight was left as missing. At the two year follow-up time point (*i.e.*, day 730), we adopted a similar strategy: (i) if weight information was available on day 730 we took that value; (ii) otherwise, if a weight measurement was observed in the +/−15 days around day 730, we carried the nearest value backward/forward; (iii) if there were at least two weight measurements, at least 30 days apart, in a 180 day interval around day 730 (*i.e.*, +/−90 days), we fit an individual-specific linear regression model to these values and interpolated to obtain a weight at day 730; (iv) otherwise, weight was left as missing. The requirement of at least two weight measurements being at least 30 days apart was set in order to prevent unstable weight extrapolation.

### 2.6. Covariates

Potential confounders of the relationship between choice of antidepressant drug treatment and weight change in time, obtained from electronic health care databases, were age, gender, having history of anxiety disorder, bipolar disorder, sleep disorder, schizophrenia and schizoaffective disorders, and smoking status at the time of initiating antidepressant treatment. The analyses were adjusted for these covariates’ status at baseline.

### 2.7. Statistical Analysis

Distributions of patient characteristics were summarized by tabulating across levels and computing percentages; continuous covariates, such as age and baseline weight, were categorized for these descriptive purposes but retained in their continuous form for modeling. These initial descriptive analyses were conducted across all unique patients in the study sample. For patients with multiple treatment episodes, a single episode was chosen at random for these descriptive analyses. 

To investigate the relationship between choice of antidepressant medication and weight change at two years we performed two sets of linear regression analyses. The first was based on the intent-to-treat (ITT) principle with outcomes at two years investigated purely on the basis of the medication prescribed at baseline (*i.e.*, regardless of subsequent treatment discontinuation and/or modification), and was *a priori* identified as our primary analysis. The second set was based on the per-protocol (PP) principle that considers the scenario in which patients continue their assigned antidepressant treatment for the full two years. 

For all analyses, fluoxetine was taken as the “referent” medication comparator (or reference group). In addition to evaluating main effects, we investigated possible effect modification of bupropion use by smoking status, since bupropion is indicated for smoking cessation and smoking cessation is known to be associated with weight gain [28]. We hypothesized *a priori* that the impact of bupropion would differ by smoking status.

Throughout, estimates of regression parameters were obtained using inverse-probability weighting [33]. For the ITT analyses, the weights were developed to account for missing weight data at baseline and/or at two years. Specifically, we considered a patient as having “complete” data if a valid weight measurement was observed in the EHR both at baseline and at two years; a patient had “incomplete” data if either or both of these measurements was missing. We then estimated a patient’s probability of having complete data as a function of risk factors observed in the EHR. Towards this, we developed a framework based on decomposing whether or not a patient had complete data into four sub-mechanisms: (i) a weight measurement was observed/imputed at baseline; (ii) the patient was continuously enrolled in the health plan until two years post-treatment initiation; (iii) a visit at an IGP clinic occurred at two years (+/−1 month) and; (iv) a weight measurement was observed/imputed at 24 months. For each of these mechanisms, a separate regression model was fit; fitted values from the four models were multiplied to provide the overall probability of complete data. For the second of these mechanisms (*i.e.*, continuously enrolled at 2 years) we modeled time-to-disenrollment using a Cox regression model; doing so permitted the use of information from patients who were administratively censored during follow-up. For the other three mechanisms, we modeled the appropriate binary outcome using logistic regression. For the PP analyses, these weights were combined with a separate set of weights, the inverse of the probability of treatment adherence throughout the two year follow-up. These probabilities were obtained from a Cox regression, which modeled time to treatment discontinuation; we obtained the probability of a patient to adhere to treatment throughout the two year follow-up. Finally, we note that all weights were truncated at the 90th percentile of their distributions to ensure stability [34]. While our primary results are based on these weighted analyses, for the sake of comparison, we also report unweighted analyses.

To ensure valid inference, we used the sandwich estimator [35] of the standard errors to account for the estimation of the inverse probability weights. Furthermore, to account for within-patient correlation (among those patients with more than one episode), we used a multiple outputation procedure [36]. Briefly, we developed multiple data sets of independent observations by selecting a single observation at random within each cluster/patient. We analyzed each resulting dataset, and combined the estimated regression parameters and standard errors according to a formula. 

Throughout, all analyses were performed in R version 3.1.1. All *p*-values and confidence intervals were two-sided with statistical significance judged at α = 0.05 level.

## 3. Results

### 3.1. Population Description

**Figure 1:**
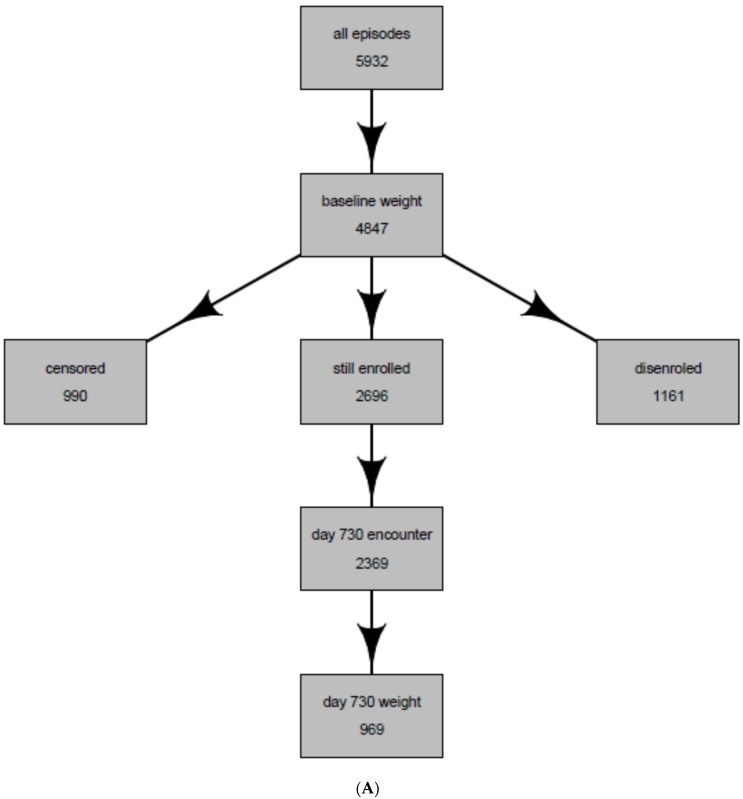

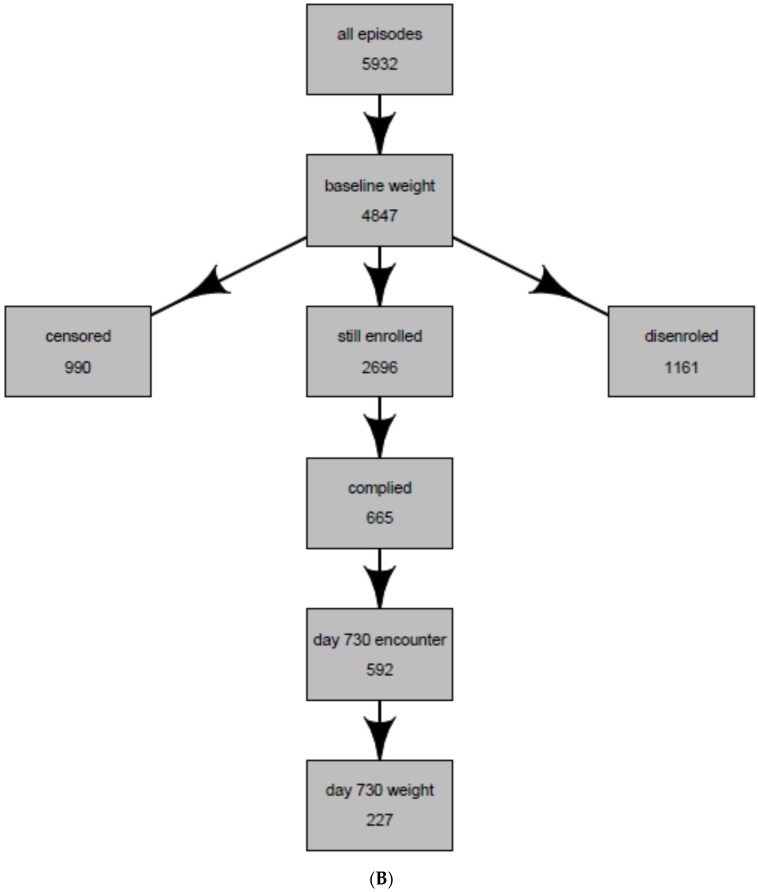
(**A**) Flow of the intent-to-treat population of antidepressant users; and (**B**) Flow of the per-protocol population of antidepressant users. Censored = patients were censored if they were using drugs or underwent procedures that have strong effect on weight change (see methods); Still enrolled = still enrolled at Group Health at day 730 (two years); Day 730 encounter = patient had an ambulatory encounter in the Integrated Group Practice (IGP); Day 730 weight = patient had a weight recorded at that encounter.

Applying our inclusion/exclusion criteria, we identified 5932 patients who initiated 6186 monotherapy antidepressant treatment episodes during the study period. Of the 6186 episodes, 1017 episodes (among 969 patients) had complete baseline weight data and two-year weight data (Figure 1A); 229 episodes (among 967 patients) had complete weight data at both time points and had remained on treatment for the full two years (Figure 1B).

From Table 1 we see that the male-to-female ratios in the ITT and PP populations are similar to that of the entire population (approximately 67%–70% female). In addition, older patients were more likely to have complete weight data at two years (43% of the ITT population was 50–65 years of age compared to 36% in the entire population) and also to have completed two years on treatment (50% were 50–65 years of age). Similarly, the heaviest patients (220–500 lbs) were more frequently in the ITT and PP populations. Smokers comprised 32% of the population of treatment initiators, while only 29% and 22% of the ITT and PP populations, respectively.

Finally, fluoxetine users were 48% of treatment initiators, and tended to be more frequently represented in both the ITT and PP populations (52% and 56%, respectively). Users of other antidepressants had slight variations in representation rates across these population groups; bupropion users were slightly less frequently represented in the ITT and PP populations than in the treatment initiators. 

### 3.2. Intent-to-Treat Analysis Results

Table 2 presents results of unweighted and inverse probability weighted ITT linear regression analyses of two-year weight change. Bupropion was the only drug that yielded a significantly different estimate of two-year weight loss when compared to fluoxetine, and then, only among patients who were non-smokers. In weighted analyses, non-smokers who initiated bupropion treatment experienced a weight change of −7.1 lbs compared to fluoxetine users who were non-smokers (95% CI: −11.3, −2.8; *p*-value < 0.01). In contrast, smokers who initiated bupropion treatment gained, on average, an estimated 2.2 lbs compared to fluoxetine users who were non-smokers (95% CI: −2.3, 6.8; *p*-value = 0.33). Mirtazapine initiators gained, on average, an estimated 11.6 lbs compared to fluoxetine users, although this difference was not statistically significant (95% CI: −2.8, 26.0; *p*-value = 0.12) likely due to the small number of mirtazapine treatment initiators in the data. All other antidepressant drug weight change estimates were not significantly different from fluoxetine, except for sertraline users, who gained 5.9 lbs compared to fluoxetine users (95% CI: 0.8, 10.9; *p*-value = 0.02).

Table 3 presents the mean baseline weight and BMI as well as the change in weight and BMI for each antidepressant medication in our study based on our ITT and IPW analysis. The mean baseline weight ranged from a low of 151.9 lbs for mirtazapine and a high of 199.1 lbs for non-smokers receiving bupropion. The mean baseline BMI ranged from 24.2 kg/m^2^ for mirtazapine to 31.5 kg/m^2^ for non-smokers receiving bupropion. The mean weight change at two years among the fluoxetine non-smokers was +4.6 lbs, while there was mean weight loss of 2.4 lbs among bupropion non-smokers. Bupropion smokers gained only slightly more weight (6.9 lbs) than fluoxetine smokers (6.7 lbs). 

### 3.3. Per Protocol Analysis Results

Table 4 presents the results of both unweighted and IPW PP analysis. In our weighted analyses, bupropion was the only drug that yielded a significantly different two-year weight change compared with fluoxetine patients, with non-smokers who initiated bupropion treatment losing an estimated 8.4 lbs (95% CI: −16.5, −0.3; *p*-value = 0.041) compared to non-smoking fluoxetine users. Smokers who initiated bupropion treatment gained an estimated 14.2 lbs (95% CI: 3.4, 24.9; *p*-value = 0.001) compared to fluoxetine non-smokers. Note that the estimated weight change in the non-smoking bupropion users is similar in the ITT and PP analysis; however, smokers are estimated to gain about 11 lbs more if treated continuously for two years compared to bupropion treated patients in the ITT analysis, most of whom did not complete two years of treatment (Table 2). Other antidepressant drug effects on body weight were smaller and not statistically significantly different than fluoxetine. There were an insufficient number of mirtazapine, duloxetine, and venlafaxine users to estimate two-year weight change for those drugs using our PP analyses.

## 4. Discussion

In this large, population-based study of antidepressant use among patients with diagnosed depression, we found that bupropion was associated with significantly less weight gain than fluoxetine among non-smokers after two years follow-up, while there was no significant difference in weight gain between bupropion and fluoxetine among smokers. Bupropion has previously been associated with weight loss in short-term (one year or less duration) studies of both depressed and non-depressed individuals [18,37]. While the mechanism of the weight-reducing effect of bupropion has not been determined, it is suspected that the dopaminergic and noradrenergic effects of bupropion play important roles in the regulation of appetite, satiety, craving, and feeding behavior [38]. The only other antidepressant medication in our study that was associated with significant weight changes at two years was sertraline, which was associated with modest weight gain compared with fluoxetine. Only the findings for bupropion were robust when we considered those patients who had continued treatment with the medications for a full two years (our PP analysis). The results are consistent with a recent 12-month study conducted by Blumenthal and colleagues involving over 22,000 adults in a single health system in New England [24].

Our study is important because antidepressants are among the most commonly prescribed medications in the United States (10.4% of adults) [9], and because there is currently no evidence to suggest that antidepressant medications differ in terms of their efficacy [26,27]. Given that bupropion is the only antidepressant associated with long-term weight loss, this medication should be the first-line drug of choice for all overweight and obese patients unless there are other existing contraindications such as a history of seizure disorder, anorexia nervosa or bulimia, or patients undergoing abrupt discontinuation of ethanol or sedatives including anticonvulsants, barbiturates, or benzodiazepines. All other antidepressant medications should be considered second-line pharmacological treatments for depression among overweight and obese patients.

Our prior research with this same population demonstrated that body weight was significantly associated with the choice of initial antidepressant medication, although the associations were weak [39]. Patients with lower BMIs were more likely to receive mirtazapine and those with higher BMIs were more likely to receive bupropion. The current study confirms that providers should strongly consider the selective prescribing of bupropion in patients who are overweight or obese. However, not all patients with depression are overweight or in need of weight loss, and, as a result, bupropion may be less appropriate for normal and underweight individuals who are nonsmokers. Similarly, in any patients who are underweight, it may be desirable to consider an antidepressant that is associated with weight gain, such as sertraline.

Our study has a number of limitations that should be mentioned. Our analysis was limited only to patients in the integrated group practice of a single large health plan; this form of analysis should be replicated in other systems. Second, in our PP analysis, we have a very small number of people actually completing the two years of treatment. We are reassured that the findings in this cohort are very similar to the larger group in the ITT analysis. However, the ITT analysis does include patients who initiated treatment but did not continue the antidepressant drug for the full two years, so the estimates of drug effects may be biased. We did not have enough patients receiving some antidepressant medications (mirtazapine, duloxetine, and venlafaxine) to estimate two year weight changes using both the ITT and PP methods. The changes in weight that we observed were clinically small for all but the bupropion users. Some second-generation antidepressants were not on the Group Health formulary and were thus excluded from our analyses (including escitalopram, fluvoxamine, and nefazodone) owing to low numbers of patients prescribed these drugs in our databases. While we excluded patients taking a number of other medications that might potentially influence the trajectory of body weight (antipsychotics, weight loss drugs), we did not look for or exclude patients who may have received metformin, lisdexamfetamine, topiramate, cyproheptadine, megestrol, or cannabinoid derivatives. Therefore our results should be interpreted with some caution as it is possible that we included some patients who received these medications while not accounting for their potential weight effects. Overall, we expect the effects of these drugs to have only a minor influence on our main result as most are rarely prescribed and have only a modest impact on weight.

In conclusion, we find that bupropion is the only antidepressant associated with long-term weight loss (although this effect is limited to non-smokers). Given similar efficacy for improvement in depressive symptoms across bupropion and other second-generation antidepressants, bupropion may be considered the first-line drug of choice for overweight and obese patients unless there are other existing contraindications.

## Figures and Tables

**Table 1:** Characteristics of the entire population of antidepressant users and antidepressant users with observable weight data at two years *.

	Entire Population of Antidepressant Users *	Population Whose Weight Was Observed at 2 years (Intent-to-Treat Population)	Population Whose weight Was Observed and Were Continuously Treated with the Same Antidepressant at 2 years (Per-Protocol Population)
Total	5932	969	227
Female, *N* (%)	4018 (67.7%)	651 (67.2%)	158 (69.6%)
Age, *N* (%)			
18–29 years	1114 (18.8%)	119 (12.3%)	17 (7.5%)
30–49 years	2691 (45.4%)	438 (45.2%)	96 (42.3%)
50–65 years	2127 (35.9%)	412 (42.5%)	114 (50.2%)
Weight, *N* (%)			
88–149 lbs.	1176 (19.8%)	200 (20.6%)	42 (18.5%)
150–179 lbs.	1111 (18.7%)	226 (23.3%)	57 (25.1%)
180–219 lbs.	724 (12.2%)	148 (15.3%)	32 (14.1%)
220–500 lbs.	1188 (20.0%)	268 (27.7%)	69 (30.4%)
No baseline weight	1085 (18.3%)	0 (0%)	0 (0%)
Body mass index, *N* (%)			
Underweight: <18.5	41 (0.7%)	9 (0.9%)	1 (0.4%)
Normal: 18.5–24.9	1217 (20.5%)	216 (22.3%)	46 (20.3%)
Overweight: 25–29.9	1324 (22.3%)	279 (28.8%)	66 (29.1%)
Obese: ≥ 30.0	1957 (33.0%)	441 (45.5%)	110 (48.5%)
No baseline BMI	1393 (23.5%)	24 (2.5%)	4 (1.8%)
Antidepressants, *N* (%)			
Fluoxetine	2842 (47.9%)	506 (52.2%)	127 (55.9%)
Bupropion	877 (14.8%)	129 (13.3%)	25 (11%)
Citalopram	1137 (19.2%)	173 (17.9%)	39 (17.2%)
Duloxetine	37 (0.6%)	8 (0.8%)	0 (0.0%)
Mirtazapine	36 (0.6%)	5 (0.5%)	1 (0.4%)
Paroxetine	245 (4.1%)	34 (3.5%)	9 (4%)
Sertraline	367 (6.2%)	47 (4.9%)	18 (7.9%)
Trazodone	281 (4.7%)	54 (5.6%)	6 (2.6%)
Venlafaxine	110 (1.9%)	13 (1.3%)	2 (0.9%)
Concurrent psychotherapy *N* (%)	680 (11.5%)	100 (10.3%)	21 (9.3%)
Comorbid conditions, *N* (%)			
Anxiety disorder	1527 (25.7%)	247 (25.5%)	49 (21.6%)
Bipolar disorder	78 (1.3%)	9 (0.9%)	4 (1.8%)
Schizophrenia	1 (0.0%)	0 (0.0%)	0 (0.0%)
Schizoaffective disorder	1 (0.0%)	0 (0.0%)	0 (0.0%)
Sleep disturbance	381 (6.4%)	80 (8.3%)	18 (7.9%)
Smoker	1923 (32.4%)	282 (29.1%)	49 (21.6%)

* The first column corresponds to characteristics of the entire population who had at least one monotherapy antidepressant treatment episode during the study period; the second column describes the characteristics of patients who were observed for weight change at two years, and were used in the intent-to-treat (ITT) analysis. The third column describes the characteristics of patients whose treatment episodes lasted at least two years, and were used in the per-protocol (PP) analysis. All covariates are measured at baseline.

**Table 2:** Estimated 2-year weight change (lbs) for users of the various drug groups compared to fluoxetine users based on the intent-to-treat analysis *.

	Unweighted Estimates			Weighted Estimates		
	Estimate	*p*-Value	95% CI	Estimate	*p*-Value	95% CI
Bupropion-non smoker	−7.6	<0.01	(−11.5, −3.7)	−7.1	<0.01	(−11.3, −2.8)
Bupropion-smoker	1.0	0.65	(−3.2, 5.2)	2.2	0.33	(−2.3, 6.8)
Citalopram	0.3	0.82	(−2.3, 2.9)	1.2	0.40	(−1.6, 4.1)
Duloxetine	−0.6	0.91	(−11.4, 10.1)	−1.0	0.88	(−13.5, 11.5)
Mirtazapine	12.7	0.08	(−1.5, 27.0)	11.6	0.12	(−2.8, 26.0)
Paroxetine	−0.5	0.84	(−5.7, 4.7)	0.8	0.78	(−5.0, 6.6)
Sertraline	3.3	0.15	(−1.2, 7.9)	5.9	0.02	(0.8, 10.9)
Trazodone	0.4	0.84	(−3.9, 4.8)	0.8	0.75	(−3.9, 5.5)
Venlafaxine	−6.7	0.14	(−15.5, 2.1)	−2.0	0.67	(−11.3, 7.3)

* Results in the left part of the table refer to the unweighted modelling that ignores selection bias, while the right side of the tables provides the inverse probability weighted (IPW) estimation results. Omnibus *p*-values for the null hypothesis “all drugs have the same effect on weight change” were 0.004 (naive analysis) and 0.009 (IPW analysis). Analyses were adjusted for age, gender, baseline weight, smoking status, and active psychotherapy.

**Table 3:** Estimated baseline weight and body mass index (BMI) and change in weight and BMI at 2 years *.

	Baseline Weight (lbs)	Baseline BMI (kg/m^2^)	Change in Weight at 2 years (lbs)	Change in BMI at 2 years (kg/m^2^)
Fluoxetine: non-smoker	191.4	30.6	4.6	0.7
Fluoxetine: smoker	186.2	29.5	6.7	1.1
Bupropion: non-smoker	199.1	31.5	−2.4	−0.4
Bupropion: smoker	194.0	30.2	6.9	1.1
Citalopram	186.6	29.8	5.9	0.9
Duloxetine	194.5	31.1	3.6	0.6
Mirtazapine	151.9	24.2	16.2	2.6
Paroxetine	189.9	30.1	5.5	0.9
Sertraline	187.5	30.0	10.5	1.7
Trazodone	188.1	29.8	5.4	0.9
Venlafaxine	183.6	29.1	2.6	0.4

* Results from the inverse probability weighted, intent-to-treat modeling analysis adjusted for age, gender, baseline weight, smoking status, and concurrent psychotherapy.

**Table 4:** Estimated 2-year weight change (lbs) for users of the various drug groups compared to fluoxetine users based on the per-protocol analysis *.

	Unweighted Analysis			Weighted Analysis		
	Estimate	*p*-value	95% CI	Estimate	*p*-value	95% CI
Bupropion: non smoker	−7.6	0.049	(−15.2, 0.0)	−8.4	0.041	(−16.5, −0.3)
Bupropion: smoker	13.3	0.016	(2.5, 24.1)	14.2	0.001	(3.4, 24.9)
Citalopram	1.9	0.49	(−3.5, 7.2)	2.9	0.32	(−2.9, 8.7)
Paroxetine	0.9	0.86	(−9.2, 11.0)	0.3	0.96	(−10.5, 11.1)
Sertraline	2.3	0.53	(−5.0, 9.6)	5.5	0.17	(−2.4, 13.4)
Trazodone	0.9	0.88	(−11.4,13.3)	−0.1	0.99	(−13.2, 13.0)

* Results in the left part of the table refer to an unweighted modelling that ignores selection bias, while the right side of the tables provides the inverse probability weighted estimation results. Omnibus *p*-values for the null hypothesis “all drugs have the same effect on weight change” were 0.21 (naive analysis) and 0.09 (IPW analysis). Analyses were adjusted for age, gender, baseline weight, smoking status, and active psychotherapy.

## Supplementary Materials

Supplementary materials can be found at http://www.mdpi.com/2077-0383/5/4/48/s1.

## Abbreviations

The following abbreviations are used in this manuscript: BMI, body mass index; GH, Group Health; IGP, integrated group practice; IPW, inverse probability weighting; ITT, intent to treat; PP, per protocol; EHR, electronic health record
