# Supplementary material for: Long-Term Weight Change after Initiating Second-Generation Antidepressants
Source: J Clin Med. 2016 Apr 13;5(4):48. doi: 10.3390/jcm5040048 (PMC4850471; doi:10.3390/jcm5040048)
Supplement: Supplementary file 1 [file jcm-05-00048-s001.docx]

**Supplementary Materials: Long-Term Weight Change after Initiating Second-Generation Antidepressants**

David Arterburn, Tamar Sofer, Denise M. Boudreau, Andy Bogart, Emily O. Westbrook,
Mary Kay Theis, Greg Simon, and Sebastien Haneuse

**T**able S1: Included and excluded medications in this study**.**

| **Included second-generation antidepressant medications** | **Excluded second-generation antidepressant medications*** | **Other excluded medications^†^** |
| --- | --- | --- |
| Bupropion, citalopram, duloxetine, fluoxetine, mirtazapine, paroxetine, sertraline, trazodone, and venlafaxine. | Escitalopram, fluvoxamine, and nefazodone | Second/third-generation antipsychotic medications (aripiprazole; clozapine; iloperidone; olanzapine; olanzapine/fluoxetine; paliperidone; quetiapine ; risperidone; and ziprasidone;)  Weight loss medications (orlistat; phentermine; and sibutramine)  Oral steroids (dexamethasone, fludrocortisone, and ) prednisone  Lithium, valproate |

* These medications were not on the Group Health formulary. ^†^ These medications were excluded because they were believed to have a strong effect on body weight.
